# Advances in Nutritional Research on Regulatory T-Cells

**DOI:** 10.3390/nu5114305

**Published:** 2013-10-28

**Authors:** Wooki Kim, Hyungjae Lee

**Affiliations:** 1 Department of Food Science and Biotechnology, Graduate School of Biotechnology, Kyung Hee University, Yongin, Gyeonggi 446-701, Korea; E-Mail: kimw@khu.ac.kr; 2 Department of Food Engineering, Dankook University, Cheonan, Chungnam 330-714, Korea

**Keywords:** nutrition, immunology, regulatory T-cells, Tregs, anti-inflammatory

## Abstract

Many clinical and animal studies have shown that certain dietary components exert anti-inflammatory properties that aid in the amelioration of chronic inflammatory diseases. Among the various proposed channels through which dietary components affect immune responses, regulatory T-cells (Tregs) are emerging as key targets for the dietary prevention of chronic inflammatory diseases. In this review, immunoregulation by Tregs is briefly described, followed by a summary of recent advances and possible applications of techniques for the study of Tregs. In addition, this review provides an overview of the current knowledge on Treg regulation by certain dietary components, including vitamins, omega-3 polyunsaturated fatty acids, and polyphenols. The caveats of previous studies are also discussed in order to highlight the distinctions between dietary studies and immunological approaches. Consequently, this review may help to clarify the means by which nutritional components influence Tregs.

## 1. Introduction to T-Cell Immunology

Higher organisms, including humans, have immune systems developed to protect the host from potentially harmful materials originating both endogenously and exogenously. In other words, immune responses can also be described as a mechanism of distinguishing “self” from “non-self” or “abnormal self”. One of a variety of immunological constituents, T-cells play a pivotal role in both innate and adaptive immune responses [1], directly by exerting cytotoxic effects on infected cells or indirectly by modulating other types of immune cells. Cytotoxic T-cells (Tc), also called cluster of differentiation (CD)8^+^ T-cells due to their characteristic expression of CD8 protein on cell surfaces, mediate the direct toxicity against infected cells.

**Figure 1:**
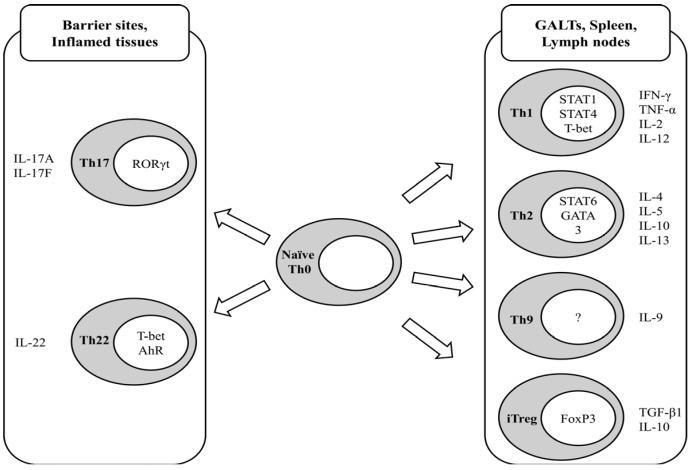
Schematic representation of CD4^+^ T-cell differentiation and characteristic cytokine production of differentiated subtypes. GALT stands for gut-associated lymphoid tissue.

Indirect immunomodulation is controlled by CD4^+^ T-cells, also called helper T-cells (Th), which regulate other immune cell types. During activation by antigen encounter, naïve (previously antigen-unexposed) CD4^+^ T-cells (Th0) are further differentiated into effector subtypes, which have been distinguished to date as Th1, Th2, Th9, Th17, and Th22 cells, depending on the milieu of tissues, type of antigen, and corresponding co-stimulating molecules on the antigen-presenting cells. The Th1 subtype, distinguished by nuclear factors STAT1, STAT4, and T-bet, produces interferon (IFN)-γ, tumor necrosis factor (TNF)-α, interleukin(IL)-2, and IL-12, resulting in the activation of effector cytotoxic CD8^+^ T-cells and innate immune cells such as macrophages [2,3]. Th2 cells, in which the nuclear factors STAT6 and GATA3 are present, secrete IL-4, IL-5, IL-10, and IL-13, which further activate B-cell transformation to plasma cells in order to provide humoral immunity through the production of antibodies. In contrast to the aforementioned systemic helper T-cells, IL-17-producing Th17 cells have been identified in local inflammation-prone sites, including the digestive tract. It has been reported that the orphan retinoic acid receptor (ROR) family transcription factor RORγt is essential for Th17 development and function [4], indicating that vitamin A, a precursor of retinoic acid, is a potential dietary modulator of Th17 cells and consequent inflammatory responses. Indeed, numerous data have indicated that Th17 cells are highly relevant to the onset and propagation of local chronic inflammation, in part by producing inflammatory cytokines, including IL-17 [5,6,7,8]. IL-22-producing Th22 cells have been demonstrated to be central to host protection against bacterial infections at barrier sites. The development of Th22 cells is dependent on the expression of the transcription factors T-bet and AhR [9]. Th9 cells were identified by the production of IL-9 [10,11], the *in vivo* significance of which has not been clearly identified, although Th9 cells are thought to be involved in allergic inflammation, anti-tumor immunity, and autoimmune inflammation (reviewed in [12]). The differentiation of CD4^+^ T-cells is summarized in Figure 1.

## 2. Regulatory T-Cells

In contrast to the aforementioned T-cell subtypes, all of which promote specific types of immune responses, regulatory T-cells (Tregs) were identified in the late 1970s by their unique ability to suppress immune responses [13]. Two distinctive regulatory mechanisms of Tregs have been discovered: direct cell-cell contact with inflammatory effector T-cells and secretion of anti-inflammatory cytokines, such as TNF-α and IL-10 [14]. As for the development of the cell lineage, Tregs develop in either the thymus (natural Tregs, nTregs) or secondary lymphoid tissues (inducible Tregs, iTregs) such as spleen, lymph nodes, and intestinal mucosa [15]. nTregs require CD28 costimulation during positive selection in the thymus in the presence of TGF-β, IL-2, and IL-15. It has been reported that Helios, an Ikaros transcription factor family member, is uniquely expressed in nTregs [16] but not in iTregs. However, this is still under debate owing to the recent report of the coexistence of both Helios^+^ and Helios^−^ nTregs *in vivo* [17]. Because of their low prevalence among CD4^+^ T-cells *in vivo*, the function of nTregs is not clear at present, but they are thought to be responsible for the suppression of inflammatory signals, either by cell-cell contact, secretion of the anti-inflammatory cytokines TGF-β1 and IL-10, or both. iTregs, however, develop under subinflammatory conditions, such as in inflamed tissues or normal intestinal mucosa with costimulation of CTLA-4 in the presence of TGF-β and IL-2. iTregs exert anti-inflammatory effects on activated effector T cells through the secretion of IL-10 and TGF-β1. Because iTregs develop from naïve CD4^+^ T-cells in the periphery lymphoid tissues, the repertoire of iTregs is specific to allergens, commensal microbiota, neoantigens, alloantigens, and self-antigens [18,19].

Through the use of immunostaining techniques with specific antibodies, iTregs were found to express CD25 molecules, a surface marker of “antigen experienced” T-cells, indicating that iTregs are not naïve but develop through certain inflammatory responses. In fact, iTregs are specifically abundant in the digestive tract, respiratory tract, and other inflammatory sites, where the influx of exogenous materials is a common occurrence. It is well known that a variety of microbiota living in the intestinal tract discharge a tremendous amount of microbial products such as lipopolysaccharides, flagellins, double-stranded RNAs, and DNA fragments rich in cytosine and guanine (CpG islands). Regulation of the immune system to properly maintain host defenses while suppressing chronic inflammatory responses to remarkable amounts of bacterial products is not yet completely understood. Recently, iTregs were recognized as one of the major constituents that regulate intestinal inflammatory disease [20,21]. Furthermore, in a mouse model with increased gut permeability resulting from the genetic deletion of *Junctional Adhesion Molecule-A (JAM-A)*, an increased influx of microbial products provoked immune responses, resulting in the development of inflammatory Th17 cells as well as anti-inflammatory iTregs [22]. Thus, an analogy is frequently drawn between iTregs and “firemen” in “burning” (*i.e.*, inflamed) sites.

## 3. *In vitro* Induction and Intracellular Staining of iTregs

As mentioned above, iTregs are unique in that they develop from the same progenitor cells as inflammatory effector T-cells. Therefore, the increasing focus on iTregs as targets for regulation of inflammation by therapeutic or nutritional means requires experimental approaches to identify iTregs in a mixed population of cells. The differentiation of CD4^+^ T-cells into a specific subpopulation is dependent on the expression of nuclear factors. The nuclear factor forkhead box P3 (Foxp3) was found to be such a key constituent, and directs the conversion of naïve CD4^+^ T-cells into Tregs [23].

**Figure 2:**
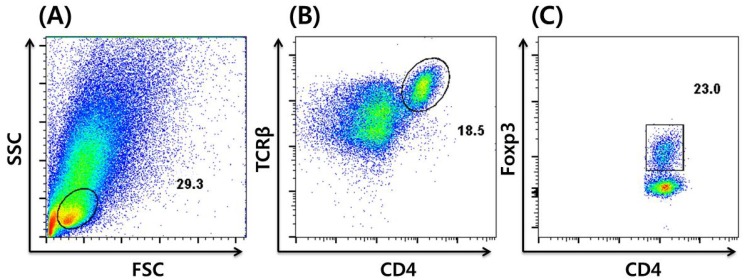
Flow cytometric analysis of Tregs by intracellular staining of Foxp3. (**A**) Lymphocytes are gated by forward scatter (FSC) *vs*. side scatter (SSC), followed by (**B**) CD4^+^ T-cell gating on CD4 and TCRβ. (**C**) Foxp3^+^ Tregs gated out of CD4^+^ TCRβ^+^ T-cells. The numbers indicate the percentage of the gated cells out of the total number of cells within the plot.

An advance in immunostaining techniques enabled the staining of intracellular nuclear factors, including Foxp3; briefly, a single-cell suspension of a cell mixture is stained using monoclonal antibodies specific to CD4 and T-cell receptor β (TCRβ), followed by cell fixation and permeabilization. After washing, a monoclonal antibody specific to Foxp3 is applied in order to stain that nuclear factor. The stained cells are analyzed by flow cytometry, followed by analysis using dedicated software. A typical characterization of Tregs by immunostaining and flow cytometric analysis of a single-cell suspension derived from murine colon is shown in Figure 2.

As for experiments testing the direct role of specific factors in Treg differentiation from naïve T-cells, *in vivo* studies are complicated by the fact that various cells interact with each other. *Ex vivo* differentiation of naïve CD4^+^ T-cells into Tregs (Ti-Treg) was made feasible by culture of the naïve cells in the presence of Treg-promoting cytokines [24]. Briefly, naïve CD4^+^ CD25^−^ T-cells are purified using surface marker-specific magnetic beads and magnetic separators, followed by culture with mitogenic antibodies (anti-CD3 and anti-CD28 monoclonal antibodies) and transforming growth factor (TGF)-β1. By following this protocol, more than 60% of CD4^+^ T-cells were converted to Foxp3^+^ Ti-Tregs in five days [24]. These *ex vivo* converted Ti-Tregs were further shown to express Foxp3 mRNA and to properly suppress effector T-cell proliferation, as assessed by real-time PCR and an intracellular dye (CFSE) dilution test, respectively. Therefore, this method of induction of Ti-Tregs may be a potent tool to investigate the direct role of specific environmental factors, including food ingredients, in the differentiation of Tregs.

## 4. Dietary Regulation of Tregs

### 4.1. Vitamins

Following dietary consumption and absorption in the intestines, water-insoluble vitamin A (all-trans-retinol) is carried by cellular retinol binding protein (CRBP) in an aqueous environment to be transported into the cytoplasm. Subsequently, retinol is oxidized to retinal by retinol dehydrogenase, and then retinal is further oxidized to retinoic acid by retinal dehydrogenase [25]. With respect to the role of vitamin A in the development of Tregs, Bai *et al*. [26] demonstrated that the population of Tregs derived from biopsies of ulcerative colitis patients increases following *ex vivo* culture in the presence of retinoic acid, a potent metabolite of vitamin A. In the same study, using a chemical (2,4,6-trinitrobenzene sulfonic acid, TNBS)-induced murine colitis model, it was further elucidated that dietary vitamin A ameliorated colitis, which is accompanied by an increased population of Tregs. Wu *et al*. [27] further reported that intraperitoneal administration of all-trans retinoic acid aided in the attenuation of airway inflammation by inducing Treg development in a model of experimental allergic asthma. Moreover, a dietary study demonstrated that retinal intervention in mice upregulated Tregs, which further assisted in the treatment of autoimmune inflammatory disorders, including rheumatoid arthritis [28]. Overall, these animal studies indicate that vitamin A and its metabolites affect populations of Tregs, thereby suppressing chronic inflammatory diseases. However, it remains unclear whether vitamin A affects Tregs directly, indirectly, or both. Indeed, Chang *et al*. demonstrated that dendritic cells also promote the generation of Tregs in response to retinoic acid, at least *in vitro* [29], exemplifying that *in vivo* models are more complicated for elucidating the mechanism of action of dietary components.

With respect to water-soluble vitamins, Kunisawa *et al*. [30] showed that Tregs express high levels of vitamin B_9_ (folic acid) receptor on their cell surfaces. Furthermore, vitamin B_9_ was demonstrated to be a survival factor for Tregs; in a vitamin B_9_-deficient culture, naïve CD4^+^ T-cells successfully differentiated into Foxp3^+^ Tregs but failed to survive. Moreover, it was found that mice fed a vitamin B_9_-deficient diet exhibited a decreased number of Tregs in the small intestine, where vitamin B_9_ is absorbed.

### 4.2. Dietary Fatty Acids

A significant quantity of data has indicated that dietary omega-3 polyunsaturated fatty acids (PUFA) may prevent or ameliorate chronic inflammatory diseases, including inflammatory bowel diseases [31,32,33,34]. These studies have identified multiple anti-inflammatory mechanisms of omega-3 PUFA: cytokine production, antagonism to omega-6 PUFA metabolism, binding to nuclear receptors as ligands, the alteration of signaling protein acylation, and the modulation of signaling platform lipid rafts in various immune cell models. With respect to CD4^+^ T-cell mediated inflammatory responses, several studies using dietary intervention with either purified omega-3 PUFA or fish oil (which is rich in omega-3 PUFA) demonstrated that CD4^+^ T-cell functions, as assessed by cytokine production and proliferation, were suppressed in both humans [35,36,37] and experimental animals [38,39].

As for the effect of omega-3 PUFA on Tregs, it was shown that the omega-3 PUFA abundant in fermented fish oil enhanced the development of Foxp3^+^ Tregs *in vivo* [40]. Moreover, intraperitoneal injection of eicosapentaenoic acid (EPA), an omega-3 PUFA, resulted in prolongation of graft survival in a murine transplant model, accompanied by an increased population of Tregs [41]. However, those studies do not conclusively demonstrate a direct function of omega-3 PUFA on the differentiation and/or function of Tregs, given the caveat that *in vivo* administration of omega-3 PUFA can affect diverse types of accessory cells. Indeed, it was found that dendritic cells cultured in docosahexaenoic acid (DHA, an omega-3 PUFA)-rich conditions facilitated the development of Tregs, at least *in vitro* [42], indicating an indirect modulation of Treg development by omega-3 PUFA. However, despite the increase in the development of Tregs, the favorable effect of omega-3 PUFA on Tregs is still controversial because it has also been observed that DHA inhibited the suppressive effect of Tregs on effector T-cell proliferations in a dose-dependent manner *in vitro* [43].

### 4.3. Dietary Polyphenols

Dietary polyphenols are well known for their antioxidant properties, which can further suppress inflammatory responses by reducing nitric oxide. In addition to their archetypal antioxidant capacities, immunoregulatory effects of select polyphenols on Tregs have been characterized as follows. Wang *et al*. [44] reported that *ex vivo* development of CD4^+^ Foxp3^+^ Tregs was induced in the presence of the flavonoid naringenin in part via activation of the aryl hydrocarbon receptor, a transcription factor. As well, increased Treg differentiation suppressed the proliferation of effector T-cells. In addition, dietary naringenin was found to suppress cholesterol-induced systemic inflammation, metabolic dysregulation, atherosclerosis, and allergen-induced airway inflammation [45,46], although it was not clear whether those inhibitory effects were Treg-dependent. In a mouse model of T-cell mediated inflammatory disease of the central nervous system, dietary intake of epigallocatechin gallate (EGCG), a catechin derivative, exerted a favorable effect, in part by increasing the Treg population in the spinal cord [47]. Furthermore, fermented grape marc (FGM) was found to promote Treg differentiation of human CD4^+^ T-cells [48].

## 5. Conclusions

Due to the relatively short history of studies on Tregs, investigations of the direct effects of a variety of dietary components on Treg conversion are limited at present (summarized in Table 1 and previously reviewed elsewhere [49]). It seems that a majority of the anti-inflammatory functional studies have been conducted *in vivo* by observing physiological outcomes, such as symptoms of chronic inflammation and accumulation of certain immune cell types. Even though *in vivo* studies are indispensable for nutritional studies, simpler model systems must be developed to dissect the detailed mechanisms by which dietary components regulate physiological systems. Therefore, further studies using more sophisticated and appropriate *in vivo* model systems are needed to draw solid conclusions. In this regard, as introduced in this review, the protocol for Treg differentiation from naïve CD4^+^ T cells could be effectively utilized to determine the direct effects of a variety of promising dietary Treg modulators present in foods.

**Table 1:** Summary of the modulation of Tregs by dietary components.

Dietary component	Function	Reference
**Vitamin A**	Increased Treg population in human biopsy culture from ulcerative colitis patients *in vitro*.	[26]
	Amelioration of TNBS-induced murine colitis model *in vivo*.	
**Vitamin B_9_**	Essential for murine Treg survival *ex vivo*.	[30]
	Increased Treg population in murine small intestine *in vivo*.	
**Omega-3 PUFA**	Enhanced development of murine Tregs *in vivo*.	[37]
	Increased Tregs in a murine transplant model *in vivo*.	[38]
	Increased ability of murine dendritic cells to convert Tregs *in vitro*.	[39]
	Decreased regulatory function of murine Tregs *ex vivo*.	[40]
**Polyphenols**		
Naringenin	Increased murine Treg population *in vivo*.	[44]
EGCG ^1^	Increased murine Treg population *in vivo*.	[47]
FGM ^2^	Increased human Treg population *in vivo*.	[48]

^1^ EGCG, epigallocatechin gallate; ^2^ FGM, fermented grape marc.
